# CrossDocker: a tool for performing cross-docking using Autodock Vina

**DOI:** 10.1186/s40064-016-1972-4

**Published:** 2016-03-17

**Authors:** Jamal Shamsara

**Affiliations:** Pharmaceutical Research Center, School of Pharmacy, Mashhad University of Medical Sciences, 91775-1365 Mashhad, Iran

**Keywords:** Autodock Vina, Cross-docking, Virtual screening

## Abstract

**Background:**

Cross-docking is an approach to find the best holo structures among multiple structures available for a target protein.

**Results:**

CrossDocker significantly decreases the time needed for setting parameters and inputs for performing multiple dockings, data collection and subsequent analysis.

**Conclusion:**

CrossDocker was written in Python language and is available as executable binary for Windows operating system. It is available at http://www.pharm-sbg.com. Some example data sets were also provided.

## Background

Structural based virtual screenings are widely used for identification of new lead compounds for specific targets that their experimental 3D structure is available (Kubinyi 2006). The successfulness of such virtual screenings greatly depends on the quality of the available 3D structure of the receptor (Pitt et al. 2013). In general, holo structures perform better than apo structures. In case of multiple available 3D structures for a receptor, selection of the best structure for pose prediction and virtual screening is an important issue (Mohan et al. 2005). They are several suggested methods for selection of the best structure (Hawkins et al. 2008). One is the selection according to the specification of the X-ray crystallography. The most prominent one is the resolution of the X-ray crystal structure. Others are R-factor and average B-factor. For example it was proposed that the quality of a crystal structure can be estimated considering resolution and R-factor (Sacan et al. 2012).

**Fig. 1:**
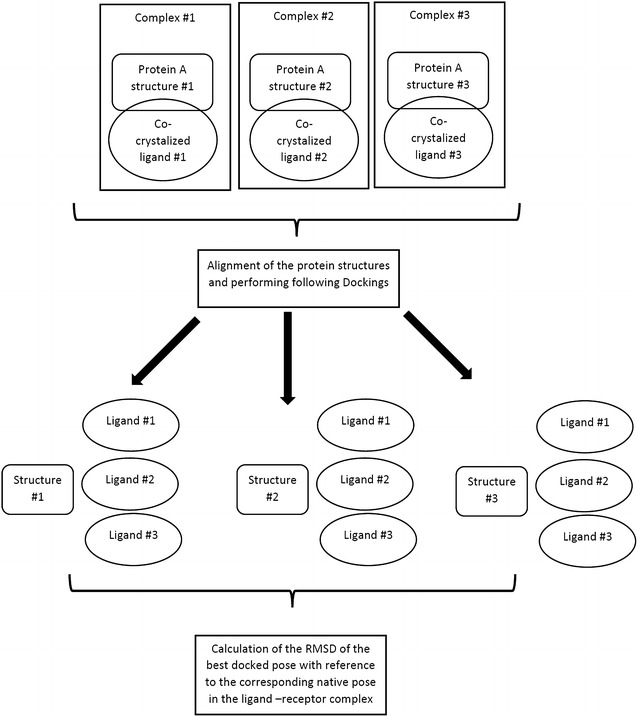
A typical workflow of a cross-docking study on a data set of three ligand-receptor complexes

However, these metrics are not absolute and it was proposed (Vinh et al. 2012; Ramezani and Shamsara 2015) that solely considering these structural parameters of an x-ray crystal structure cannot properly predict the performance of a 3D structure in virtual screenings, especially in case of receptors with flexible active site. Flexible receptors could exhibit either intrinsic or induced flexibility (Chandrika et al. 2009). Different ligand can induce different conformational changes in the active site residue of a receptor upon binding. Thus, a single ligand-receptor complex solved in the presence of a specific ligand may have a lower affinity for another ligand (with different scaffold). In the other words, the chemical characteristics (size, functional groups, etc.) of the co-crystalized ligand is also important for the applicability of a flexible 3D structure in virtual screenings (Ramezani and Shamsara 2015; Zhang et al. 2014). For example, a flexible binding pocket which is arranged to have interactions with a small ligand (in a crystal structure) cannot easily accommodate binding of larger ligands (in a virtual screening). These can be indirectly determined by a retrospective virtual screening on a predefined set of active compounds and decoys or a cross-docking (Fig. 1) study. It was suggested that the structures that have the best ability to dock non-native ligands with lower RMSD with reference to the crystalled pose of the ligands are probably more successful in prediction of binding pose (Zhang et al. 2014) of the ligands correctly and virtual screenings (Ramezani and Shamsara 2015).

On another hand, the performance of docking algorithms and scoring functions are varying for different targets (Cheng et al. 2012; Warren et al. 2006; Shamsara 2014). This can also be assessed by self and cross-docking. If the proper pose of a ligand in the active site is well modeled by a method and ranked higher among other possible poses it can be an indicator of suitability of the method for a given target protein.

In this paper we described CrossDocker which can significantly accelerate performing multiple dockings, data collection and subsequent analysis.

## Implementation and preparation of inputs

The whole process of cross-docking was implemented as a computer program using the python language. As a case study 12 holo 3-hydroxy-3-methylglutaryl-coenzyme A (HMG-CoA) reductase structures were retrieved from PDB (PDB codes: 1HW8, 1HW9, 1HWJ, 1HWK, 1HWL, 2Q1L, 2Q6B, 2Q6C, 2R4F, 3BGL, 3CCT, 3CCW, 3CCZ, 3CD0, 3CD5, 3CD7, 3CDA and 3CDB). The retrieved holo crystal structures from the PDB were aligned before docking to make the RMSD calculation possible after dockings. The protein alignment can be done by several open source or commercial tools such as PyMol or Chimera. All the structures were aligned by PyMol using align command. After structural alignment, the first two chains (A and B) of the each PDB file, adenosine-5′-diphosphate (ADP) and ligands were retained. The co-crystalized ligand and protein were saved as two individual files. CrossDocker detects the corresponding ligand-receptor by their file names. The name of the protein can be anything but should end with “-p” and the name of the native ligand should be same as the protein name and ends with “-l”. Thus, each Receptor/ligand pairs were separated into two pdb files with same root-name (e.g. their PDB codes) and different suffix, “-p” and “-l” respectively. CrossDocker reads structures in mol2, pdb or pdbqt formats and for receptors it considers all cofactors and coenzymes as a part of receptor molecule and removes all water molecules. Then ligands and receptors were divided into two separate folders. There is a configuration file (config.txt) that the path to the receptors and ligands folders can be set there. Some parameters for the run of Autodock Vina (Trott and Olson 2010) can also be set in this file (see the explanations in config.txt file) such as grid box dimensions and maximum number of binding poses to be generate for each dock (coordination will be set automatically by CrossDocker according to the coordination of the ligand). The initial conformation of the ligands was randomized by CrossDocker using “randomize_only” option of Autodock Vina prior to docking to avoid bias toward conformation of ligands in the crystal structure.

## Results

**Fig. 2:**
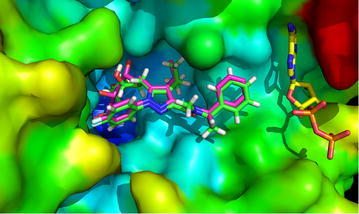
Docked pose (*green*) of the ligand of 2R4F PDB code in the active site of HMG-CoA reductade structure with PDB code 1HWJ. The experimental pose of the ligand in PDB code 2R4F is shown in *magenta*. ADP is shown in *yellow* color. The calculated RMSD is 0.96 Å. The atoms in the 1HWJ active site were color coded by their B-factors. *Blue* is for low B-factor and *red* is for high B-factor value. Higher B-factor may indicate flexibility of the residues (inaccuracy in crystallography for some part of the protein also causes the higher B-factor)

“Output.xlsx” contains RMSD and energy calculated for each pose of each docked ligand in every receptor. The best RMSD for each dock and its docking energy is reported in “Output_the_best_RMSD.xlsx”. “Table_the_best_RMSD.xlsx” contains best RMSD obtained for each ligand for each receptor. It shows the number with conditional formatting: green represents the 10 ‰, yellow the 50 ‰ and red the 90 ‰. The values for self-dockings are in bold faces. Thus, the interpretation of the results would be very easy. The average RMSD for each 3D structure is reported. The number of docks with RMSD <2.0 Å is another parameter that is reported in “Table_the_best_RMSD.xlsx” file. “Table_the_best_energy.xlsx” contains the best energy obtained for each ligand docked in each receptor. “Table_RMSD_for_the_best_energy.xlsx” includes the RMSD that is obtained for a docked pose of a ligand which has a best docked energy among generated modes by Autodock Vina. In the most cross-docking studies the best RMSDs (which can be found in Table-RMSD.xls file) were considered by authors. However, it seems that the calculated RMSD for the docked pose with the lowest energy which are reported in “Table_energy_for_the_best_RMSD.xlsx” can also be important for analysis, because in a typical virtual screening the docked poses with lowest energy are only considered for analysis. Tables 1 and 2 shows contents of the “Table_the_best_RMSD.xlsx” file and “Table_RMSD_for_the_best_energy.xlsx” generated by CrossDocker from HMG-CoA reductase data set. Figure 2 shows reliability of the docked pose of a ligand with reference to the native conformation in the active site of the structure with PDB code 1HWJ.

**Table 1:**
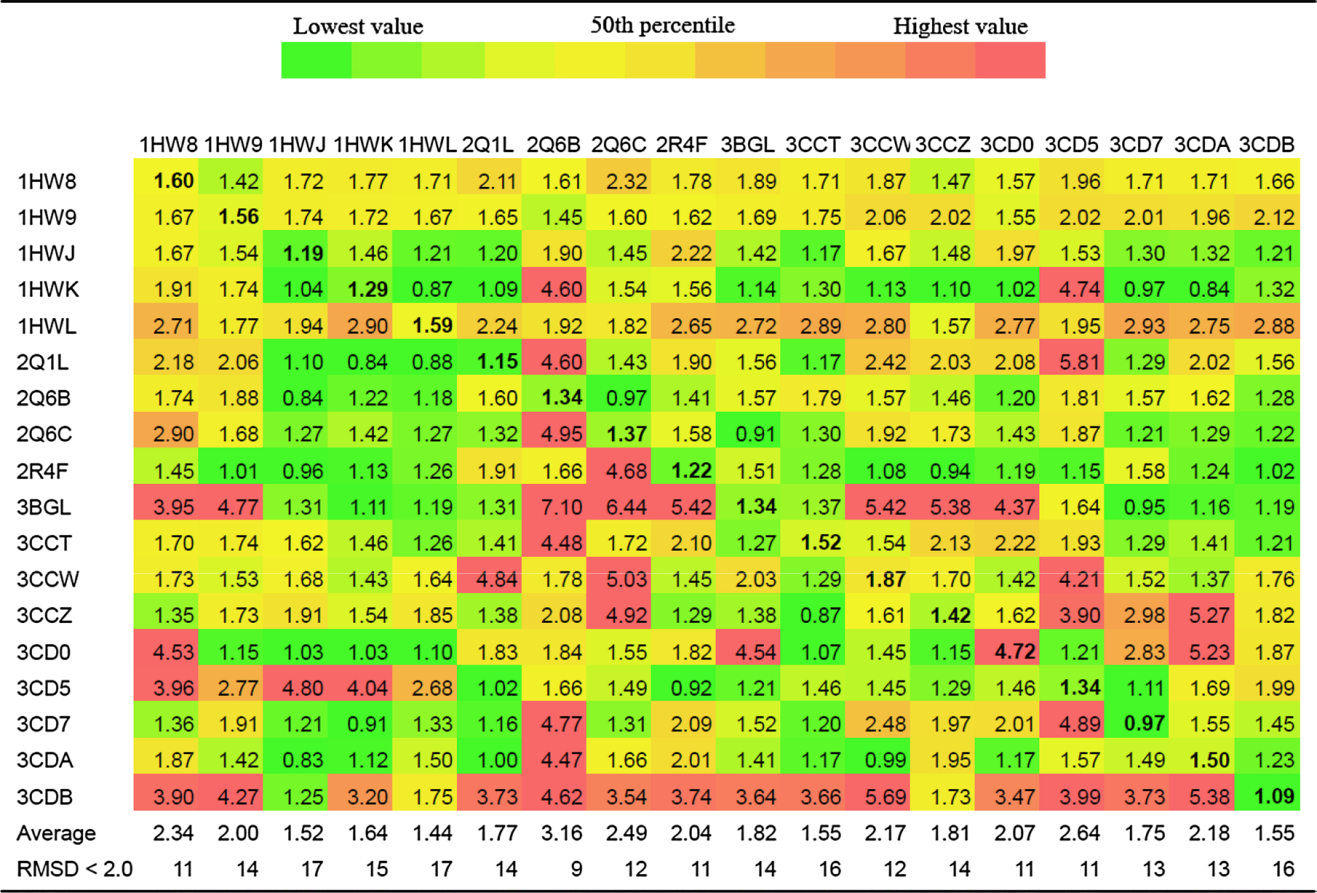
RMSD of each ligand pose which has smallest RMSD docking to each PDB code protein was shown

Receptors are in columns while co-crystalized ligands are in rows. The average of the calculated RMSDs for a receptor is presented. The number of successful docks (RMSD <2.0) is also presented for each receptor in the last row of the table. The RMSDs of self-dockings are in *bold* faces

**Table 2:**
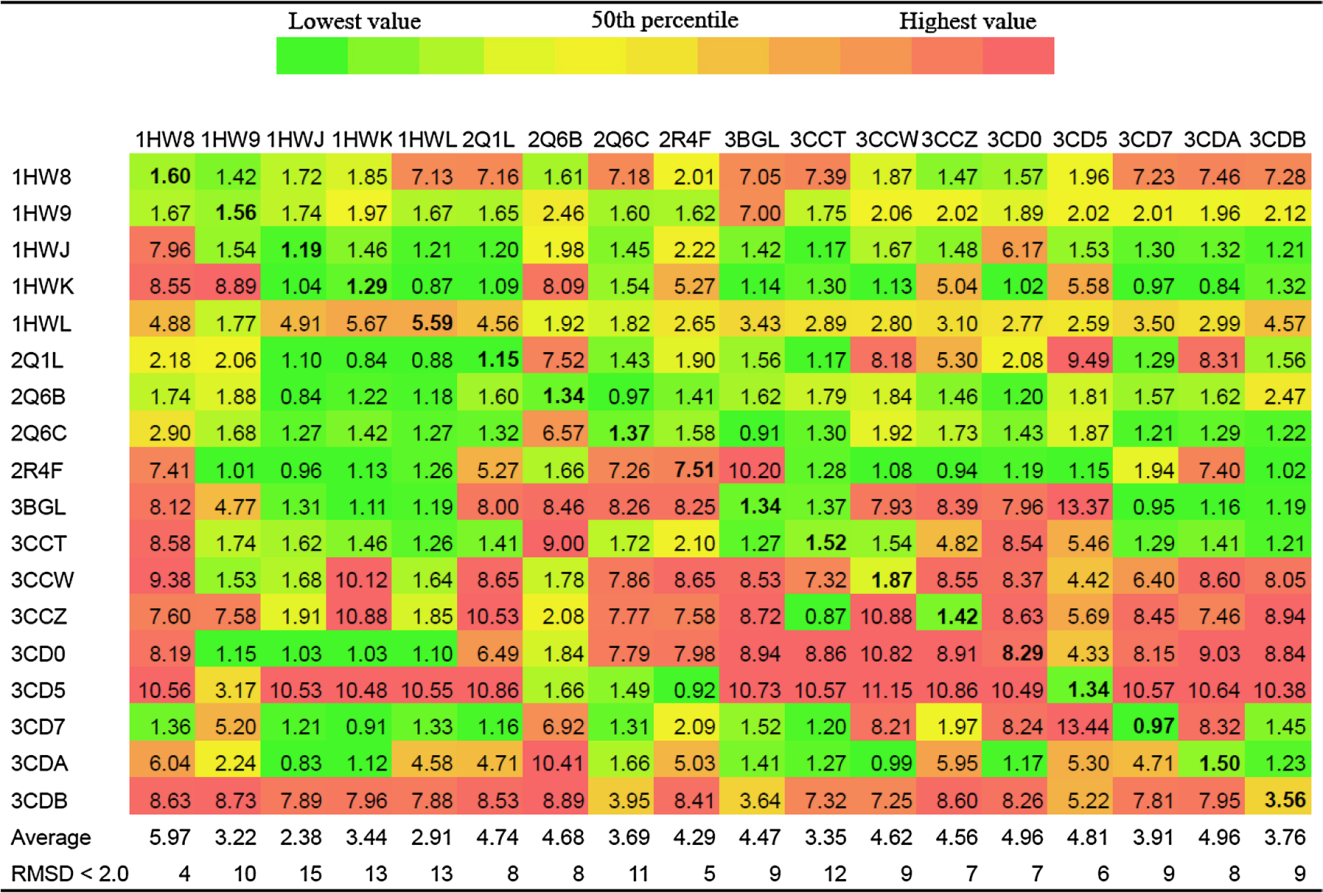
RMSD of each ligand pose which has smallest energy docking to each PDB code protein

Receptors are in columns while co-crystalized ligands are in rows. The average of the calculated RMSDs for a receptor is presented. The number of successful docks (RMSD <2.0) is also presented for each receptor in the last row of the table. The RMSDs of self-dockings are in *bold* faces

## Discussion and conclusions

CrossDocker provides a good opportunity to perform cross-docking easily on a series of 3D structure of a same receptor and subsequent data collection and analysis. As it was suggested previously the structures with lower average RMSD and/or higher number of docked poses with RMSD <2.0 Å have higher probability for good performance in virtual screening study and pose prediction (Vinh et al. 2012; Ramezani and Shamsara 2015; Zhang et al. 2014). Thus the reported results by CrossDocker can be used to achieve the best structures for computational drug design studies. Furthermore, if the most of the calculated average RMSD for a specific target is high and/or self-docking RMSDs are also high, it can indicate poor performance of Autodock Vina on a specific target and vice versa. In some cases, it can be improved by increasing exhaustiveness parameter in configuration file that would also increase the computation time. In the example above the performance of Autodock Vina on the set of HMG-CoA reductase structures was reasonable with default setting (exhaustiveness = 8) (see Fig. 2 and calculated self-docking RMSDs in Tables 1, 2). According to the results of the obtained best RMSDs, structures 1HWJ, 1HWL, 3CCT and 3CDB would be more promising to use for binding pose prediction and virtual screening studies. As the docking poses with the lowest energy normally get more attention in the analysis step of a docking study the contents of the output table entitled “Table_RMSD_for_the_best_energy.xlsx” should also be considered for selection of the best PDB structure. Thus, according to Table 2, 1HWJ is the best one among those four structures. In this paper the applicability of CrossDocker was shown. CrossDocker was written in Python language and available as executable binary for Windows operating system. All examples input and output files as well as CrossDocker are available at http://www.pharm-sbg.com.

## Availability and requirements

- Project name: CrossDocker project;
- Project home page: http://www.pharm-sbg.com;
- Operating system(s): Windows;
- Programming language: Python;
- Other requirements: Openbabel 2.3 or higher.
